# Design of Controllable Novel Piezoelectric Components for Microfluidic Applications

**DOI:** 10.3390/s18114049

**Published:** 2018-11-20

**Authors:** Elingas Cekas, Giedrius Janusas, Asta Guobiene, Arvydas Palevicius, Andrius Vilkauskas, Sigita Ponelyte Urbaite

**Affiliations:** 1 Faculty of Mechanical Engineering and Design, Kaunas University of Technology, Studentu str. 56, LT-51424 Kaunas, Lithuania; elingas.cekas@ktu.lt (E.C.); arvydas.palevicius@ktu.lt (A.P.); andrius.vilkauskas@ktu.lt (A.V.); sigita.ponelyte@ktu.lt (S.P.U.); 2 Institute of Materials Science, Kaunas University of Technology, Barsausko str. 59, LT-51423 Kaunas, Lithuania; asta.guobiene@ktu.lt

**Keywords:** microresonator, microfluidics, piezoelectric, chemical composition, periodical microstructure

## Abstract

This paper reviews recent investigations and achievements in the design of controllable functional components for improving microfluidic systems, its effectiveness, and functionality. The main purpose was to design novel microstructures with piezoelectric properties (microresonators), which enable one to control the effectiveness of fluid flow in micro-hydro-mechanical devices for biomedical/biochemical purposes. Controllable properties were obtained by incorporating different types of binders in a piezoelectric ceramic matrix (lead zirconate titanate): polyvinyl butyral (PVB), poly methyl methacrylate (PMMA), and polystyrene (PS). The change in chemical composition of PZT helps to manipulate the piezoelectric characteristics, surface morphology, mechanical properties, etc., of the designed microfluidic element with the microstructure in it.

## 1. Introduction

In the past two decades, microfluidic systems have been in high demand among researchers and industries. It is one of the promising future technologies for handling a small volume of fluids in microchannels for chemical, medical and biological applications in various fields. Designing effective microfluidic systems enables decreasing the analysis time, reducing the sample dose, increasing throughput ability, making it portable, and extending its integration in different fields. Passive and active microfluidic components (valves [1,2], pumps [3,4,5], etc.) and platforms (centrifugal [6], pressure-driven systems [7,8], droplet-based microfluidic systems [9], etc.) have been designed. However, despite the huge selection of microfluidic systems [10], some main drawbacks are still unsolved or they are not as effective as they should be: the ability to unlimit the manipulation of small liquid plugs in microchannels, to increase the flow of high viscosity fluids, and even to reduce air bubbles between two plugs. This research paper covers the design of novel piezoelectric microstructures employed as microresonators, which could ensure more effective flow of microfluids when the system is excited by vibrations.

Inorganic piezoelectric materials have received great attention over the past few decades due to their simple fabrication process, small mode volume, and high quality factors [11,12]. These materials (PZT, ZnO, AlN, etc.) offer a number of unique benefits compared to other materials/ceramics and are widely used in the development of microelectromechanical systems (MEMS) as filters [13,14], switches [15], generators [16], micro-pumps [17], etc. They cover many different approaches as microarrays, DNA sequencing, sample preparation and analysis, cell separation and detection, or environmental monitoring. The main advantages are small amounts of sample and reagent, less time consumption, lower cost, and high throughput [18,19,20]. Here, the polymers have been used with a number of fabrications techniques like hot embossing, injection molding, casting, laser ablation, etc.

A microresonator in the design process of an effective microfluidic system is a fundamental part and requires an effective potency to differentiate and process multivariable data [21]. Many fabrication processes exist [22,23,24], but piezoelectrically-actuated or -sensed microresonators are ideal for a large-scale integration to form an array on a single sensing or actuating platform in MEMS. Specific functionalities defined by microresonator design conditions and application areas may be manipulated by incorporating different types of binders in a piezoelectric ceramic matrix. Depending on the application, various dopants and binders are used to tailor the properties of interest. In this research paper, the main focus is given to the design of a controllable and effective microresonator employed in a microfluidic system, which may overcome the earlier mentioned drawbacks. Thus, lead zirconate titanate (PZT) was chosen as the main piezoelectric ceramic for microresonators design because of its exceptional properties [25] and biocompatibility. The addition of various binders allows modifying some of these properties, i.e., to lower dissipation factor, to increase piezoelectric coefficients, dielectric constant, loss, etc. Thus, chemicals (or binders) such as polyvinyl butyral (PVB) [26], polymethyl methacrylate (PMMA) [27], and polystyrene (PS) [28], have shown strong characteristics in microfabrication processes, i.e., strong binding, high adhesion, toughness, flexibility, etc. This interaction of these materials with lead zirconate titanate (PZT) allows changing the main microresonators properties such as the surface morphology, chemical, optical, electrical, and mechanical properties, to enhance its functionality, performance, and to simplify the design and fabrication process.

**Figure 1:**
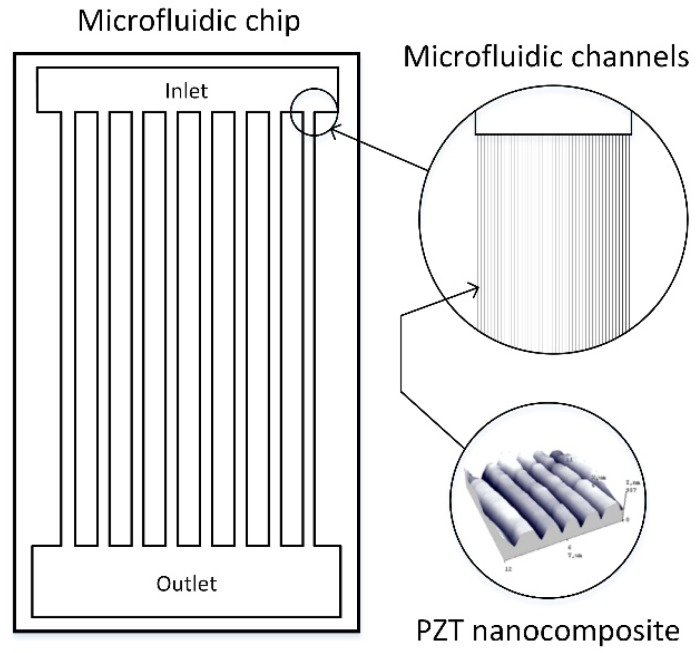
Schematic view of a microfluidic microchip and close-up of the channels, as well as the lead zirconate titanate (PZT) nanocomposite location.

This research paper covers recent progress in the fabrication of microfluidic channels made of PZT thin films (Figure 1).

Microfluid control methods using acoustic manipulation are well known in today’s scientific world, where micro-channels contain the excited standing and travelling acoustic waves. A more effective flow of microfluids is ensured by vibration methods, which excite the generated walls of the micro-hydro-dynamical system. For this to happen, the thin PZT films are integrated into the microchannels of the micro-dynamical systems, thus ensuring more effective functionality [29]. A rather simple approach was found for effective design in optimizing the surface, chemical, and electrical properties. The impact of different binders in the novel PZT microresonators’ coating properties was analyzed and described. Thus, three unique microresonators made of PZT composite material with polyvinyl butyral (PVB), polymethyl methacrylate (PMMA), and polystyrene (PS) were fabricated. These samples were examined with a scanning electron microscope (SEM) and Fourier transform infrared spectroscopy (FTIR) for the evaluation of their surface and chemical composition and a Keyence laser for the evaluation of the piezoresistive characteristics. The microhardness of the designed microresonators was evaluated with a dynamic microhardness meter (measuring the Martins and Vickers microhardness) and with a scratch tester. The obtained results from multiple sets of investigated data were used to observe the changes of the properties using different types of binders in PZT.

## 2. Materials and Methods

### 2.1. Microresonator Formation Using Three Different Binders

The fabrication of the novel PZT material started with powder preparation. This composite material was designed by mixing PZT milled powder with 3 different binders in proportions of 80% of PZT and 20% of binder. Binders used for sample fabrication were polyvinyl butyral (PVB) in benzyl alcohol, polymethyl methacrylate (PMMA) in benzyl alcohol, and polystyrene (PS) in benzyl alcohol.

An oxalate/hydroxide co-precipitation method was used for the synthesis of the lead zirconate titanate [Pb(Zr_0.52_Ti_0.48_)O_3_] nanopowder. The materials used were lead (II) acetate Pb(CH_3_COO)_2_, titanium butoxide Ti(C_4_H_9_O)_4_, zirconium butoxide Zr(C_4_H_9_O)_4_ (80% solution in n-butanol), oxalic acid dihydrate C_2_H_2_O_4_·2H_2_O, 25% ammonia solution, and deionized water.

Eight-point-two-six grams of lead (II) acetate were dissolved in 100 mL of deionized water. Thirty two grams of oxalic acid dihydrate were dissolved in 500 mL of deionized water in another glass beaker, and the solution was heated to 50 °C. Five-point-one grams of titanium butoxide and 7.65 g of zirconium butoxide 80% solution were added dropwise to the oxalic acid solution, and the reaction mixture was stirred vigorously until a clear yellow solution was obtained. Afterwards, the lead acetate solution was poured into a solution of titanium and zirconia alkoxides, and the pH of the reaction mixture was adjusted to 9 ÷ 10 by adding the 25% ammonia solution; the reaction was continued for 1 h. The white amorphous precipitate of PZT precursors was filtered under vacuum, washed with deionized water and acetone, and dried at 100 °C for 12 h. The dry powder was calcinated at 1000 °C for 9 h. Finally, the obtained PZT powder was milled and used for preparation of a screen-printing paste by mixing with the 30% solution of polyvinyl butyral (PVB) in benzyl alcohol, polymethyl methacrylate (PMMA) in benzyl alcohol, and polystyrene (PS) in benzyl alcohol. The amount of the components was calculated to get 80% of PZT and 20% of binders in the dry coating (e.g., 0.83 g of 30% PVB solution for 1 g of PZT powder). The viscosity of the paste was adjusted to 40 ± 5 Pa·s with benzyl alcohol (Brookfield Viscometer, ABZ spindle, 10 rpm, 25 ± 1 °C).

The paste was applied to copper foil substrates by conventional screen printing with three different types of polyester monofilament screens: 32/70, 48/70, and 140/34 meshes. The coatings were dried in an electric oven at 100 °C for 30 min.

The obtained specimens were of high quality and showed strong PZT dominance [30].

### 2.2. Evaluation Microresonators’ Coating Properties

#### 2.2.1. SEM for Surface Morphology and EDS Analysis

The structural and chemical compositions of the formed novel piezoelectric material were evaluated using a scanning electron microscope (SEM) FEI Quanta 200 FEG, equipped with the energy dispersive x-ray spectrometer (EDS) detector XFlash 4030 from Bruker. Samples were investigated under a controlled pressure water steam atmosphere. The maximal achievable resolution for high-vacuum (<6 × 10^−4^ Pa) was 0.8 nm, for low-vacuum (10–130 Pa) 1.5 nm and for extended vacuum mode (10–4000 Pa) 1.5 nm. The EDS detector allows detecting elements from boron (atomic number 5) to americium (atomic number 95). The chemical analysis can be determined at the chosen point, along the line, or the distribution on the surface; the mapping can be performed. A modern 30 mm^2^ area solid state drift detector was cooled with a Peltier element and provided 133 eV (at Mn K) energy resolution at 100,000 cps. Moreover, the X-ray spectroscopy method allows analyzing energy distributions, i.e., the energy differences between the various quantum states of a system and the probabilities that the system jumps between these states.

#### 2.2.2. FTIR for Chemical Composition Analysis

FTIR (a system SPECTRUM GX 2000 RAMAN, PerkinElmer, Waltham, MA, USA) was used for the investigations of changes in chemical composition when three different binders were used with PZT. The chemical compounds of the samples were examined. The diapason of the FTIR spectrum was 10,000–200 cm^−1^.

#### 2.2.3. Keithley Meter Scanner for the Piezoresistive Characteristics’ Evaluation

**Figure 2:**
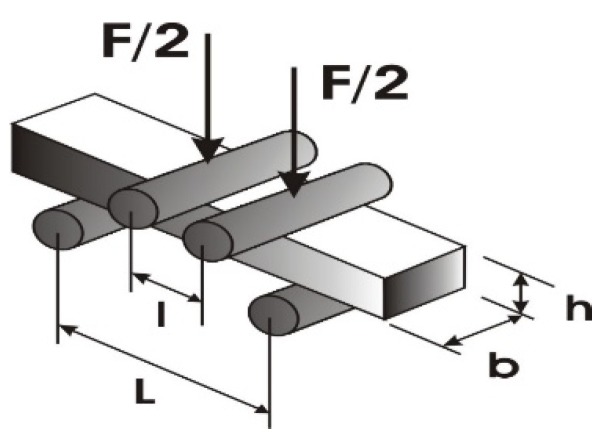
Schematic force application for analysis of the piezoresistive characteristics.

For the evaluation of the microresonators’ piezoresistive characteristics a Keithley meter scanner (Tektronix, Bracknell, UK) was employed. The experimental setup consisted of a piezoelectric energy harvester applying single hits, excitation, a measurement system, and data acquisition, i.e., this method investigates the changes of resistance and/or voltage when force F is applied to the surface of the microresonator, and after it is released (the reverse piezoelectric effect) (Figure 2). The applied mechanical energy to the surface of the coating was converted into electrical energy due to its deformation. This investigation allows examining the working conditions and comparing three different binders in PZT and the effect on the piezoresistive characteristics of a microresonator.

A gauge factor (*GF*) (ratio of relative change in electrical resistance *R* versus mechanical strain *ε*) here was calculated according to the given formula below [31]:

(1) $$GF=\frac{\Delta R/R}{\Delta L/L}$$

The mechanical strain *ε* was then calculated [31]:

(2) $$\varepsilon =\frac{\Delta l}{l}=\frac{3F\left(L-l\right)}{2bh^{2}E}$$

Here, Δ*R* is the resistance change due to strain, *R* is the initial resistance, Δ*L* is the change in length, *L* is the original length of the wire or foil, and Δ*L*/*L* is the unit strain to which the specimen is subjected, while *h* is the specimen height and *b* the specimen width.

#### 2.2.4. Triangular Displacement and PRISM for Dynamic and Electric Characteristics’ Analysis

**Figure 3:**
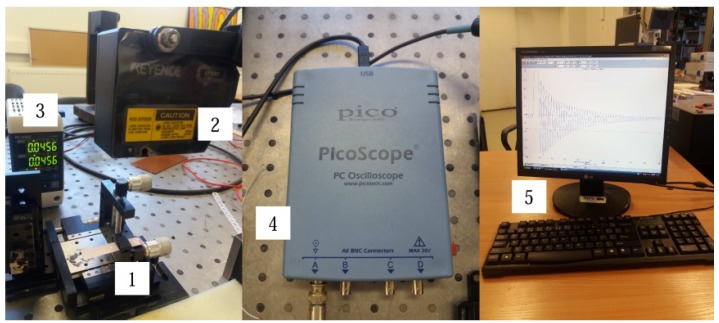
Experimental setup for dynamic and electric characterization of specimens: 1, specimen holder; 2, LK-G82 sensor head; 3, LK-G3001PV control block; 4, PicoScope oscilloscope; 5, computer.

**Figure 4:**
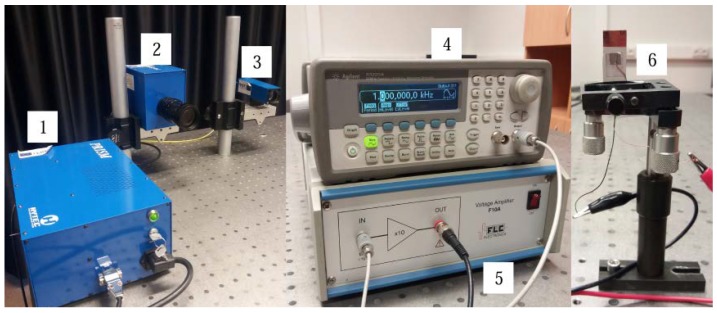
Electronic speckle pattern interferometry system PRISM: 1, control block; 2, video head; 3, illumination head; 4, generator; 5, amplifier; 6, specimen.

The dynamic and electric characteristics of the multilayer specimens were investigated using a laser triangular displacement sensor LK-G3000 (Keyence, Itasca, IL, USA). The measured vibration amplitude and generated electrical potential were collected with a USB oscilloscope PicoScope 3424 (Pico, Cambridgeshire, UK) (Figure 3). The resonant frequency was analyzed with an electronic speckle pattern interferometry system, PRISM (Hytec, Los Alamos, NM, USA) (Figure 3) [32]. It was used to investigate the mechanical response of the multilayer specimens upon electrical excitation. Green laser light (532 nm, 20 mV) was used for the measurements. In Figure 4, the laser beam (3), directed at the object (6), called the object beam and the other beam the reference beam, goes directly to the camera (2). Laser light (3) is scattered from the object and collected by the camera lens (2), which images the object onto the CCD camera sensors. The image is then sent from the camera to the computer, where it is analyzed with PRISM DAQ software.

#### 2.2.5. VSWR for Sheet Resistance

The sheet resistance of thin films was measured with the VSWR (voltage standing wave ratio) meter P2-67. It may indicate the degree of mismatch between a transmission line and its load and evaluate the effectiveness of impedance matching efforts. The VSWR measurement range was 1.1–5.0, VSWR (±5 K + 5)%, attenuation (±0.05 A × 0.75) dB, and frequency 17 GHz.

#### 2.2.6. HM 2000S Meter for Microhardness Measurement

The microhardness of the samples was measured using a dynamic microhardness meter with base HM 2000S (Fischer, Lymington, UK), which determines numerous mechanical and elastic properties of materials and thin films. Simple construction and sample positioning, as well as high quality components make this meter suitable for measuring material parameters such as Martens hardness, Vickers hardness, and elastic modulus of indentation or creep.

#### 2.2.7. Micro-Scratch Tester MST^3^ for Adhesion Failure Characterization

A micro-scratch tester MST^3^ was used to characterize the adhesion failure of the designed coatings. Linear scratching used the Rockwell-type diamond indenter (AD-295) of a 200-μm radius. The starting load was 0.02 N with 0.02 N steps to the end load of 6 N with a loading rate of 11.96 N/min. This technique was used to determine the adhesion of the coatings to ensure a sufficiently long lifetime of the microresonators’ surface adherence to the substrate.

## 3. Results

### 3.1. Structure and Chemical Composition

**Figure 5:**
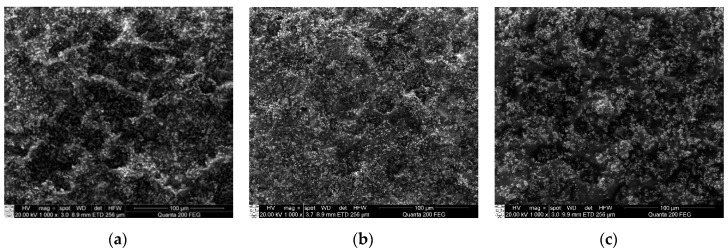
SEM views of samples with different binders mixed with PZT: (**a**) PVB, (**b**) PMMA, and (**c**) PS.

In this research, three samples of microresonators made of PZT and three different binding materials (PVB, PMMA, and PS) were fabricated and investigated. The SEM view of each sample is presented (Figure 5). The surface of the first sample, PZT with PVB binder, was rather smooth with small pileups of 10–20 μm in diameter observed on top of the surface (Figure 5a). The sample made of a PZT layer with PMMA binder was smooth, and very small pileups of less than 5 μm diameter were observed on the surface (Figure 5b). The third sample made of PZT with PS binder was also smooth with some small pileups of 5–15 μm in diameter (Figure 5c). Finally, the results showed that a binder PMMA loaded in PZT matrix might be the origin of its irregular shape, nucleation, and growth in the solution, thus forming the smaller grain group and smoother surface, compared to the PVB/PZT and PS/PZT samples.

**Figure 6:**
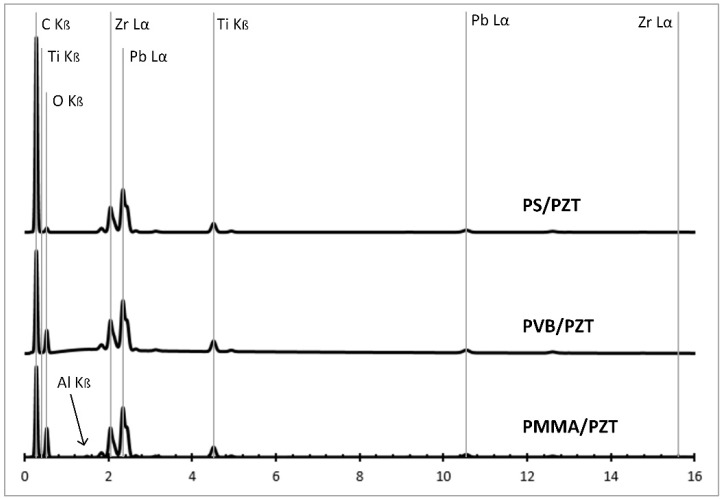
Energy dispersive spectrum of the PMMA/PZT, PVB/PZT, and PS/PZT samples.

The chemical composition of the samples was evaluated using an energy-dispersive (ED) spectrometer with a pulse height analysis. Here, an electrical charge was produced when incident X-ray photons caused ionization to occur. In the registered energy dispersive spectrum, the x axis represents the X-ray energy in Channels 1 to 6 from 0 to 6 keV, and the y axis represents the number of counts per channel up to 18 cps/keV. In all three samples, five main elements were defined: carbon (C), oxygen (O), titanium (Ti), zirconium (Zr), and lead (Pb). The energy dispersive spectrum of all samples is given in Figure 6 below.

**Figure 7:**
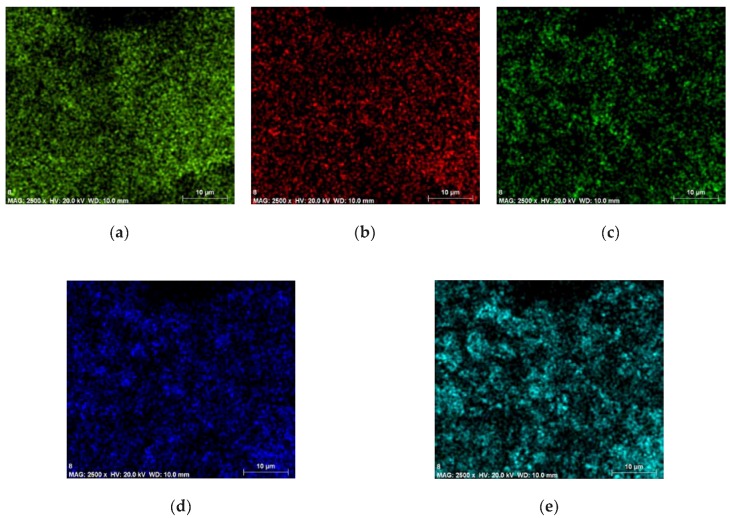
Maps of the distribution and relative proportion (intensity) of the PVB/PZT sample.

From the PVB/PZT sample ED spectrum (Figure 6) C K_β_ energy resolution, the peak was specified at about 0.2 keV. For the O K_β_ energy resolution, the peak was specified at about 0.5 keV. For the Ti K_β_ energy resolution, the peak was specified at about 0.3 keV and 4.5 keV. For Zr L_α_, the peak was achieved at 2.1 keV and 15.8 keV. For Pb L_α_, the peak was achieved at 2.4 keV and 10.5 keV. Thus, the map of the distribution and relative proportion (intensity) of defined elements over the scanned area is presented in Table 1 and Figure 7.

**Figure 8:**
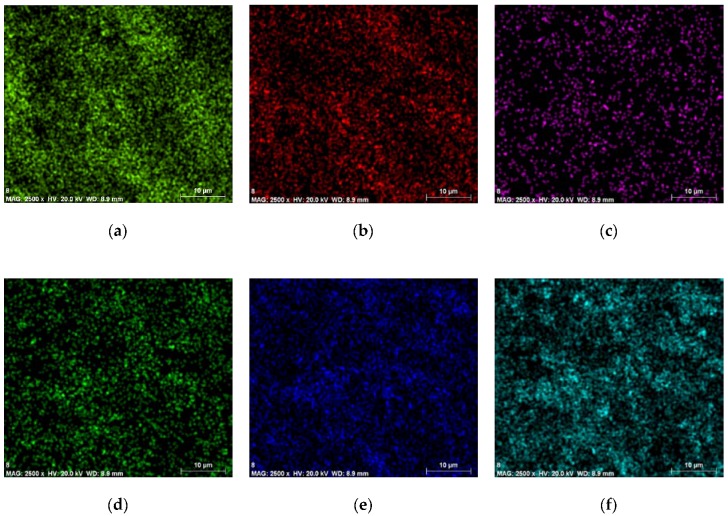
Maps of the distribution and relative proportion (intensity) of the PMMA/PZT sample.

From the PMMA/PZT sample ED spectrum (Figure 6) C K_β_ energy resolution, the peak was specified at about 0.2 keV. For the O K_β_ energy resolution, the peak was specified at about 0.5 keV. For the Ti K_β_ energy resolution, the peak was specified at about 0.4 keV and 4.5 keV. For the Al K_β_ energy resolution, the peak was specified at about 1.4 keV. For Zr L_α_, the peak was achieved at 2.1 keV and 15.8 keV. For Pb L_α_, the peak was achieved at 2.4 keV and 10.5 keV. Thus, the values were almost identical to the PVB/PZT sample, except that the aluminum element appeared. The map of the distribution and relative proportion (intensity) of defined elements over the scanned area and chemical characterization of the sample are presented in Table 2 and Figure 8.

**Figure 9:**
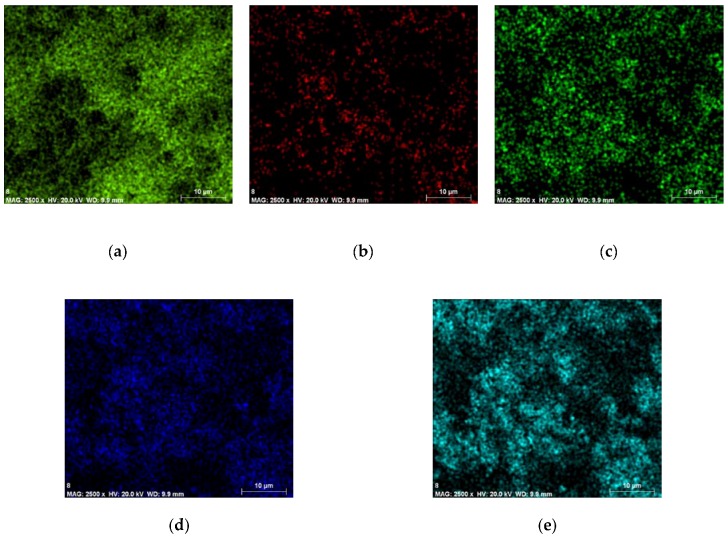
Maps of the distribution and relative proportion (intensity) of the PS/PZT sample.

The PS/PZT sample showed five main elements registered in the ED spectrum (Figure 6). The element C K_β_ energy resolution peak was specified at about 0.2 keV. For the O K_β_ energy resolution, the peak was specified at about 0.4 keV. For the Ti K_β_ energy resolution, the peak was specified at about 0.4 keV and 4.5 keV. For Zr L_α_, the peak was achieved at 2.1 keV and 15.7 keV. For Pb L_α_, the peak was achieved at 2.4 keV and 10.6 keV. Thus, the values were almost identical to the PVB/PZT and PMMA/PZT samples. The map of the distribution and relative proportion (intensity) of defined elements over the scanned area and chemical characterization of the sample are presented in Table 3 and Figure 9.

Thus, each element had its defined positions of the characteristic peak in the piezoelectric sample corresponding to the transitions in its electron shell. Chemical analysis shows that five main elements were dominant in all three samples: C, O, Ti, Zr, and Pb. Only in the PMMA/PZT sample, one more dominating element, Al, appeared. Moreover, the intensity value was dependent on the exciting X-ray’s intensity, on the X-ray detector’s efficiency, on the energy spectrum, and on the geometry of the investigated sample.

**Figure 10:**
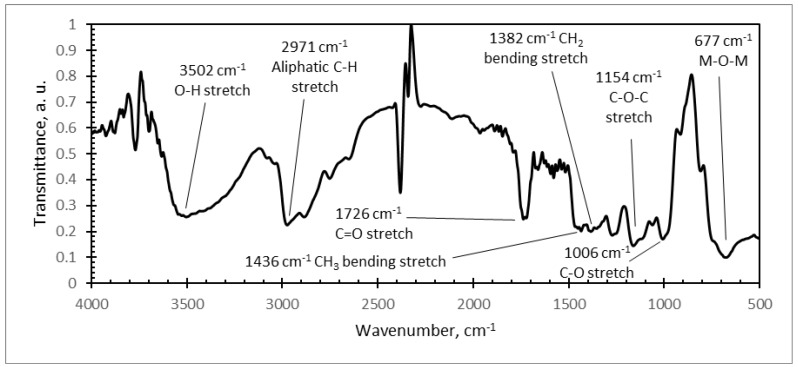
Infrared spectra of: PVB/PZT coating with a weight of 80% PZT.

**Figure 11:**
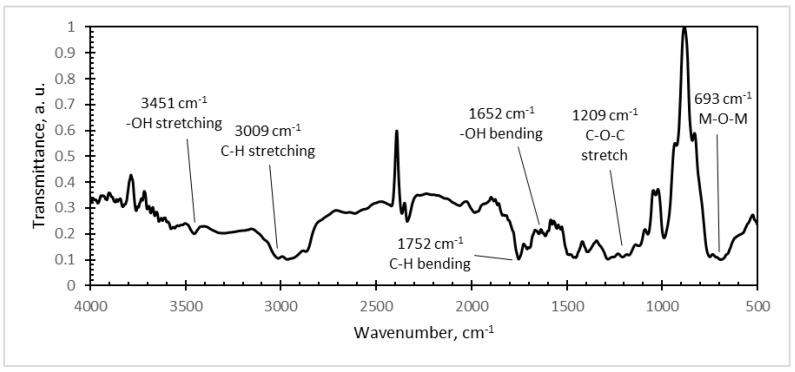
Infrared spectra of: PMMA/PZT coating with a weight of 80% PZT.

**Figure 12:**
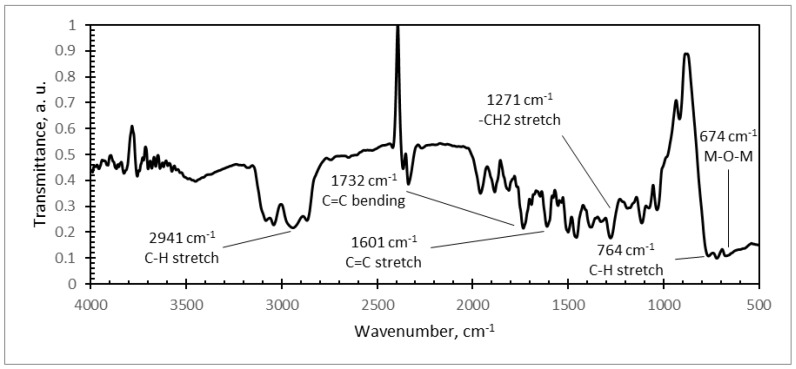
Infrared spectra of: PS/PZT coating with a weight of 80% PZT.

Further, FTIR analysis of different microresonator samples was carried out (Figure 10, Figure 11 and Figure 12). The typical FTIR absorbance spectrum is at 4000–500 cm^−1^.

In the FTIR spectra of the PVB/PZT sample (Figure 10), strong and broad absorption peaks were observed at 3502 cm^−1^ (O–H stretch), 2971 cm^−1^ (C–H stretch), 1726 cm^−1^ (C=O stretch), 1436 cm^−1^ (CH_3_ bend), 1382 cm^−1^ (CH_2_ bend), 1154 cm^−1^ (C–O–C stretch), and 1006 cm^−1^ (C–O stretch). The entire array of these peaks corresponds to the FTIR absorbance spectra of polyvinyl butyral (PVB) [33].

Strong and broad absorption peaks were observed at 3451 cm^−1^ (–OH stretch), 3009 cm^−1^ (C–H stretch), 1752 cm^−1^ (C–H bend), 1652 cm^−1^ (–OH bend), and 1209 cm^−1^ (C–O–C stretch) in the PMMA/PZT FTIR spectra (Figure 11). The entire array of these peaks corresponds to the FTIR absorbance spectra of polymethyl methacrylate (PMMA) [34].

PMMA/PZT FTIR spectra are given in Figure 12. Absorption peaks were observed at 2941 cm^−1^ (C–H stretch), 1732 cm^−1^ (C=C bend), 1601 cm^−1^ (C=C stretch), 1271 cm^−1^ (–CH_2_ stretch), and 764 cm^−1^ (C–H stretch). The received results correspond to the FTIR absorbance spectra of polystyrene (PS) [35].

In infrared spectra of all three samples, the absorption bands in diapason of 4000–1000 cm^−1^ confirmed the presence of polymers. It is obvious that PZT strongly absorbs at 700 cm^−1^, making identification of the 511 cm^−1^ band difficult for composites with ceramic contents over 20 wt.%. A wide and strong peak observed in the range of 800–550 cm^−1^ corresponds to the M–O–M bonds (M is metal) of PZT (e.g., Ti–O, Ti–O–Ti, Zr–O, and Zr–O–Zr) [36]. It may be indicating that with increasing PZT content, the PZT peak remains dominant, making the presence of binders’ peaks weak.

### 3.2. Piezoresistive Characteristics of Microresonators

Piezoresistive characteristics’ analysis of the samples was performed with a Keithley meter scanner. The width and height of the sample were 24 and 0.5 mm, respectively. The distance between the acting force points was 42 mm and 24 mm, designated as L and l. All of the different binders had the same Young’s modulus of 350 GPa. According to Figure 2 given above, the following experimental data were obtained and are presented in Table 4.

The PVB/PZT coating possessed a negative gauge factor of −139.5; the PMMA/PZT gauge factor was 1151; and the PS/PZT coating possessed the highest negative gauge factor of −3458.8. A negative polarity of the gauge factor indicates that the resistance decreases with the increasing applied strain.

**Figure 13:**
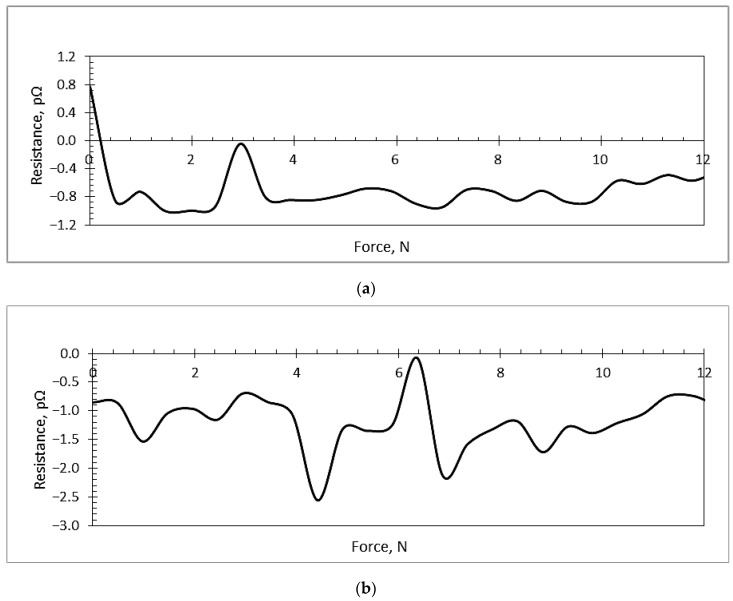

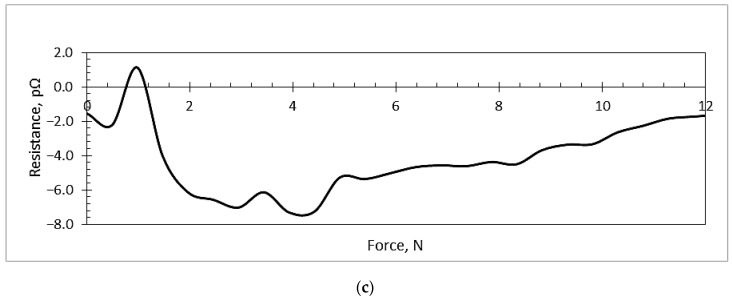
The force-resistance relationship: (**a**) PVB/PZT coating, (**b**) PMMA/PZT coating, and (**c**) PS/PZT coating.

The force-resistance relationship in all three samples (Figure 13) was linear or ascending from a certain point. For the PVB/PZT coating, this relationship became linear from ~3 N force and up (Figure 13a). Increasing force, only small changes of resistance were observed. For the PMMA/PZT coating, the resistance dramatically dropped at ~4.2 N force and then started increasing with increasing force (Figure 13b). For the PS/PZT coating, the resistance dropped till the force reached ~7 N and then started to linearly increase (Figure 13c). Thus, this piezoelectric film had an obvious turn-on threshold, i.e., a force that must be present before the resistance dropped to a value below 7 pΩ, where the relationship became more linear. The results showed that the resistance of the piezoelectric coating was highly dependent on the type of binder. All three experiments were held 15 times each with the same environmental conditions. Figure 13 shows only the average of all measurements. 

Another important factor when designing piezoelectric materials is the value of capacitance. When a piezoelectric material operates well below the resonant frequency, it behaves as a capacitor. The value of capacitance depends on the area and thickness of the coating, as well as the material properties. In this research, the capacitance of the piezoelectric coatings was estimated by [37]:

(3) $$C\approx n\cdot \epsilon_{33}\cdot \frac{A}{d_{s}}$$

where *C* is capacitance (F), *n* the number of coating layers, *ε*_33_ the dielectric constant (As/Vm), *A* the electrode surface area of a layer (m^2^), and *d_S_* the distance between the individual electrodes (layer-thickness) (m). Calculation results of each microresonators capacitance are given in Table 5 below.

Therefore, the capacitance of a piezoelectric coatings constructed of a 70 μm-thick layer was almost the same in all three samples; however, the highest capacitance was observed in the PMMA/PZT coating.

**Figure 14:**
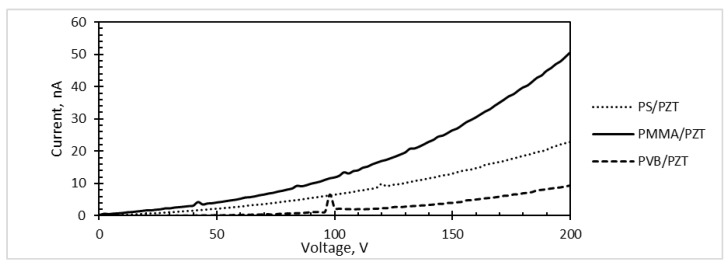
The current–voltage characteristic curve.

Further, the Keithley meter was used to evaluate the current-voltage (*I*-*V*) characteristic of each designed piezoelectric coating by sourcing the voltage from 0–200 V (2 V step) (Figure 14).

The current-voltage characteristic was defined as the relationship of the current *I* through the device and the voltage *V* across its terminals. Each experiment was done 10 times, five for each terminal. Calculations involved stepping the voltage level across a device from one level to another and measuring the resulting currents in an operation (a sweep). Thus, while sweeping a voltage from 0–200 V, the measured current of PS/PZT linearly increased up to ~23 nA and for PMMA/PZT up to ~51 nA, while PVB/PZT had a very low current increase up to 0.94 nA (Figure 14). The obtained results correspond to those found in the literature [38,39]. These results suggest making the microresonators from the PMMA/PZT material in order to increase the performance of the device, i.e., PMMA/PZT gives a significant increase in the current intensity.

**Figure 15:**
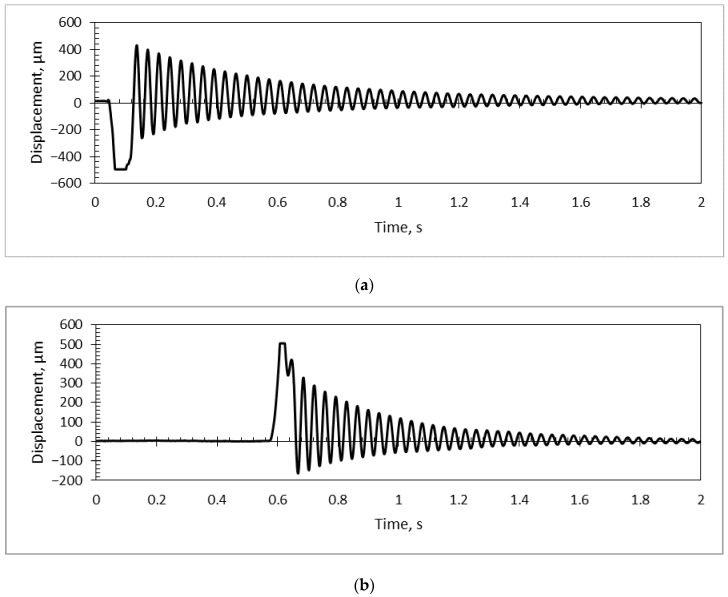

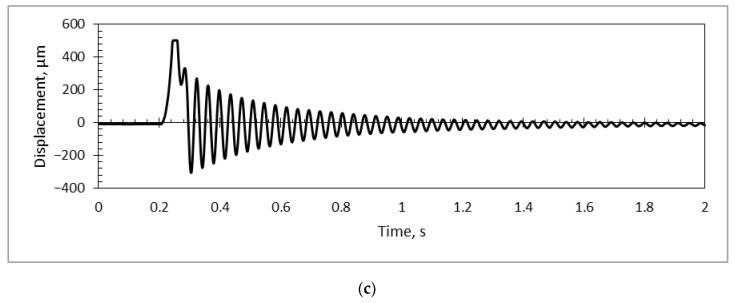
Vibration response of the (**a**) PVB/PZT, (**b**) PMMA/PZT, and (**c**) PS/PZT coating.

Using the PicoScope system together with a simulation model in COMSOL Multiphysics (for calculations of the first vibration mode), a dynamic analysis was performed for each sample, suspending a mass on a pendulum and releasing it to hit the sample, then stopping the pendulum after contact. A vibrational response of the PS/PZT coating to a mechanical impulse was registered (Figure 15).

For evaluation of the main factors of the microresonators’ quality, a value of damping ratio and Young modulus were defined (Table 6). Damping (either constructional or environmental) is one of the factors imposing an upper limit on the quality of the microresonator [40], i.e., improving the negative damping feedback, its quality is ameliorated, as well. The Young modulus defines the stiffness of the coating, i.e., stress and strain relationship in the material. Using the obtained experimental results, the main properties of each microresonator were calculated (Table 6).

The highest Young’s modulus value was obtained for the PMMA/PZT coating (6.3 GPa), while usage of PS and PVB led to a lower stiffness of 5.3 and 3.9 GPa, respectively. Since 80% of the designed material consisted of PZT nanoparticles and only 20% of binding material (PVB, PMMA, or PS), this allowed reducing the Young modulus approximately 10 times. The obtained experimental results were in good correlation with the results founded in the literature: the Young’s modulus of PMMA was 6.3 GPa [41] and PS 5.3 GPa [42], and PVB had the lowest modulus of elasticity of 3.9 GPa [43].

**Figure 16:**
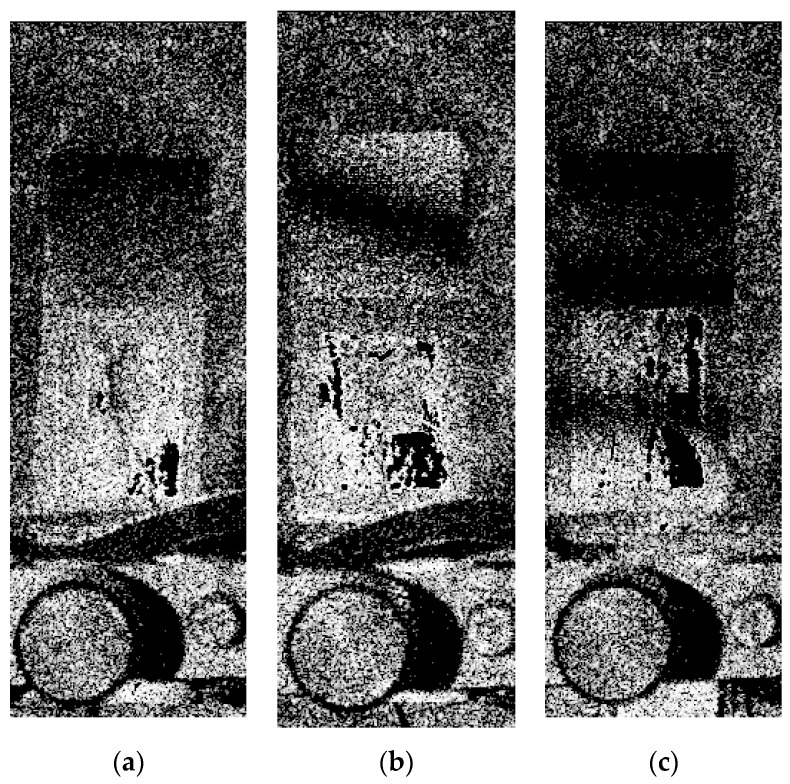
Holographic interferograms of the (**a**) PVB/PZT, (**b**) PMMA/PZT, and (**c**) PS/PZT microresonators.

Using the PRISM system, holographic interferograms together with resonant frequencies of the designed microresonators were obtained (Figure 16).

The vibration amplitude of the PVB/PZT microresonator was 100 nm at a 114-Hz resonant frequency when 100 V of voltage were applied. For PMMA/PZT, the vibration amplitude was 164 nm at a 114-Hz resonant frequency and for PS/PZT 366 nm at 114 Hz.

**Figure 17:**
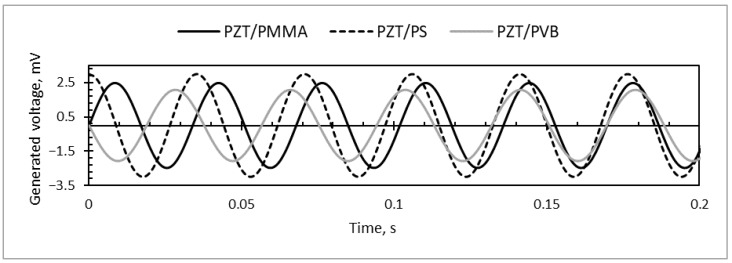
Generated voltage by periodically-excited specimens.

When the direct piezoelectric effect for further investigations of the designed microresonators was applied, the results showed that periodically-excited specimens were able to generate electrical potential due to the piezoelectric effect of the PZT material (Figure 17). The highest value of the harvested electrical energy was registered in the PS/PZT microresonator (up to 3 mV of generated voltage). The lowest result was 2.1 mV, indicated for PVB/PZT, and PMMA/PZT generated up to a 2.5-mV voltage.

The sheet resistivity of each coating was measured by the VSWR meter P2-67. Each sample was placed between a waveguide open end and a λ/4 jumper. The waveguide inner cross-section was 16 × 8 mm^2^. Thus, the results showed that each sample’s measured sheet resistance was higher than 20,000 ohms per square (Table 7). These measurements characterize the uniformity of the conductive coating for quality assurance.

### 3.3. Microhardness of Microresonators

Microhardness measurements were performed with a dynamic microhardness meter with base HM 2000S. Each sample was measured 10 times with a maximum indentation depth of 4 μm. Measurements were performed gradually under the maximum load of 200 g. Martins microhardness (HM) and Vickers microhardness (HV) were measured (Table 8).

**Figure 18:**
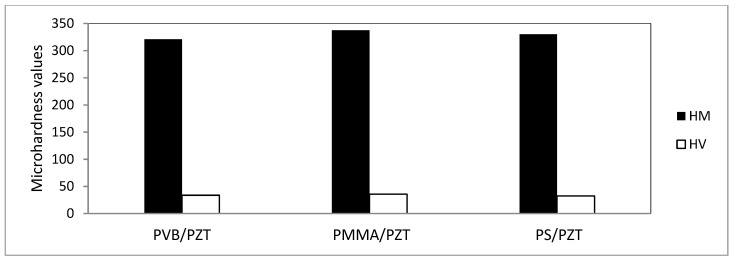
Microhardness of the microresonators.

Results showed, that there was a very slight difference in the Martins and Vickers microhardness between the different coatings, i.e., HM differed by about 17 N/mm^2^ and HV only by ~3 (Figure 18). However, the microresonators’ film with PMMA/PZT showed more elastic deformation than the other materials.

**Figure 19:**
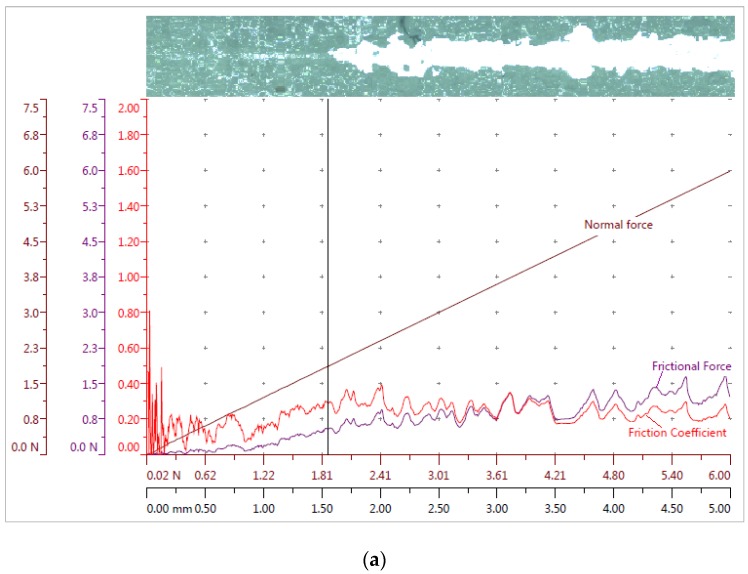

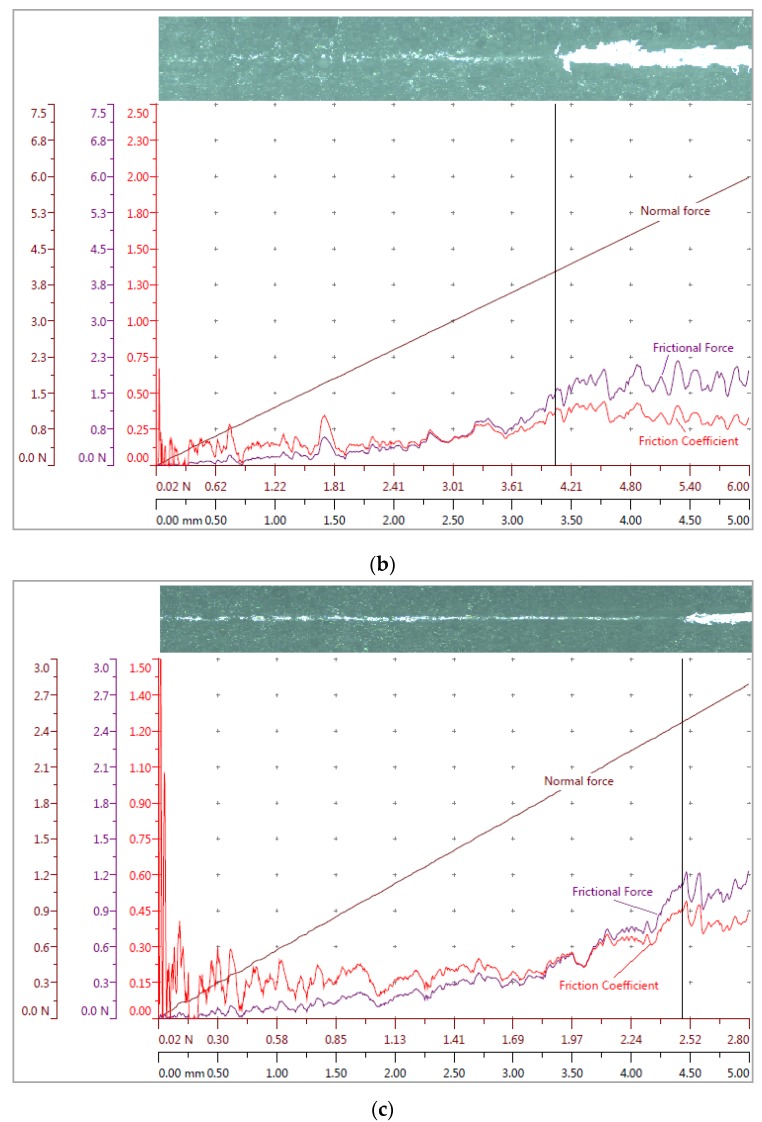
Data recorded during the scratch test: frictional force, normal force, friction coefficient, and the corresponding panorama image of (**a**) PVB/PZT, (**b**) PMMA/PZT, and (**c**) PS/PZT coatings.

During the scratch test, a critical load together with the coefficient of friction, frictional, and normal forces of the microresonators surface were identified. The failure of the coating was determined by optical microscopy, acoustic emission, and variation in penetration depth. A scratch test on a microresonator with an ~2 μm-thin coating was performed for each coating (PVB/PZT, PMMA/PZT, and PS/PZT) with a 0.2-mm radius diamond and a 5-mm scratch length. The normal load was increased over 30 s from 0–6 N. Four repetitions for each coating were done, and the main data recorded during the scratch tests are shown in Figure 19.

The obtained results imply that the PMMA/PZT coating was almost twice as hard compared to the other two, i.e., the average critical load was 3.988 N, meaning a very resistant coating to the scratch damage. The PVB/PZT coating was rather soft and not that resistant with a critical load of 1.865 N. The PS/PZT average critical load was 2.556 N. In the given panorama images, an area corresponds to the critical failure (critical load, marked vertical line in Figure 19) in the scratch track. The coating was completely removed as the substrate (brighter area) was reached during the test. The obtained critical load values showed excellent repeatability in the test results (Table 9).

The scratch testing method belongs to these new characterization techniques, which is used for adhesion measurement of functional surface coatings. The advances in adhesion testing (including panorama and prescan/post-scan procedures) now readily allow for testing of the adhesion and scratch resistance of microresonators surface coatings, which are used for the improvement of osseointegration or for reducing friction and wear. This test allows for fast and simple characterization of new functional coatings, of their adhesion, and scratch resistance in a well-controlled manner.

## 4. Microfluidic Application

**Figure 20:**
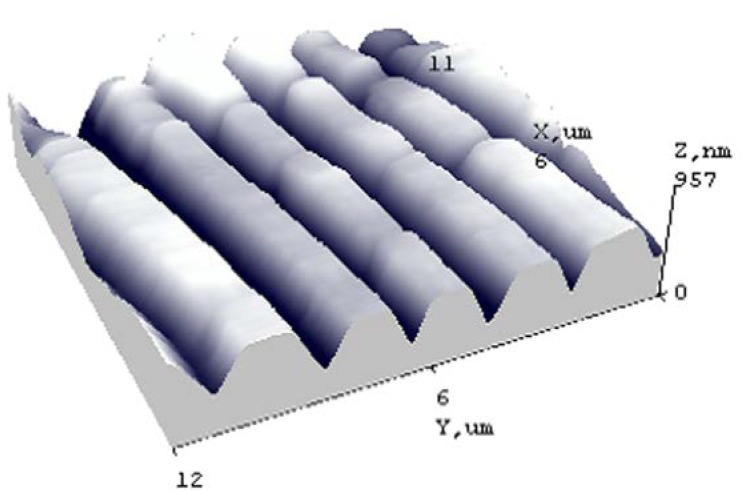
3D view of the periodical microstructure hot embossing imprinted in PMMA/PZT.

An important advantage of the designed microresonators is their integration in a microfluidic device, forming a periodical microstructure in PMMA/PZT, which may exhibit vibrations when excited. Two outputs of the system are then available: electrical signal and diffraction efficiency changes. Due to the simultaneously working electrical and optical measurement techniques, the effectiveness of the microfluidic device increases significantly. Hot embossing was used to form a lamellar periodical microstructure (period 4 μm, depth 574 nm) in the PZT nanocomposite material at a temperature of 150 °C, 5 atm pressure, and a holding time of 10 s (Figure 20).

**Figure 21:**
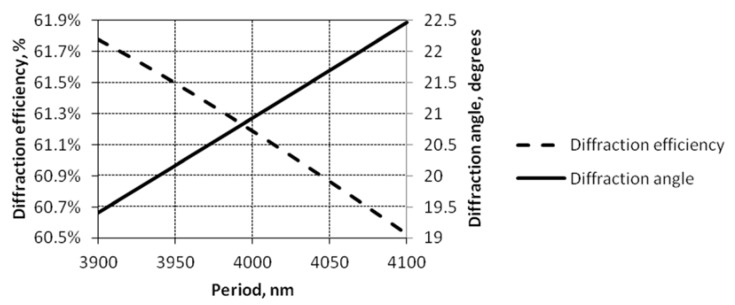
Diffraction efficiency and diffraction angle versus period of periodical microstructure.

The periodical microstructure was formatted using the composite material, which enables one to control the optical parameters, as well as the mechanical properties of the microfluidic device. Figure 21 presents the dependence of the reflected diffraction efficiency of the first order diffraction maxima and that its angle of diffraction on the electrical signal caused period changes. Change of 0.1% of diffraction efficiency and about a 15° diffraction angle were registered when the period changed by 10 nm.

A microchannel with a periodical microstructure in a microfluidic system may effectively increase its functionality.

One of the possible future applications was analyzed, which implements a syringe pump drive together with the microfluidic chip. In the range of the very high frequency (VHF) of 122 MHz, the obtained results have shown a 0.2-μL/s fluid velocity. Further analysis and additional applications will be executed in the following research.

As stated above, the microstructure may be excited with vibration methods, which ensure a more effective flow of microfluids. In this manner, it is possible to increase the flow rate of fluids, to reduce air bubbles when two liquid plugs are mixed, to reduce sample consumption, to decrease analysis time, etc.

## 5. Conclusions

A rather simple approach was found for an effective design to optimize the surface, chemical, and electrical properties of the microresonator. The impact of the different binders (PMMA, PS, and PVB) for novel PZT microresonators’ coating properties was analyzed in brief.

Three microresonators were designed. Their chemical composition showed that each element had its defined positions of the characteristic peak in the piezoelectric coating corresponding to the transitions in its electron shell. Chemical analysis showed that five main elements were dominant in all three microresonators: C, O, Ti, Zr, and Pb. Only in PMMA/PZT, one more dominating element, Al, appeared.

Investigation of the piezoresistive characteristics showed that PVB/PZT coating possesses a negative gauge factor of −139.5. The PMMA/PZT gauge factor was 1151. The PS/PZT coating possessed the highest negative gauge factor of −3458.8, where a negative polarity of the gauge factor indicated that the resistance decreased with the increasing applied strain. Moreover, PMMA/PZT and PS/PZT piezoelectric films have an obvious turn-on threshold. Investigating microresonators in the reverse piezoelectric effect, when a voltage from 0–200 V was applied, the measured current of PS/PZT linearly increased up to ~23 nA and for PMMA/PZT up to ~51 nA, while PVB/PZT had a very low current increase up to 0.94 nA. The resistance of the piezoelectric coating was highly dependent on the type of binder. Therefore, the capacitance of a piezoelectric coatings constructed of a 70 μm-thick layer was almost the same in all three samples; however, the highest capacitance was observed in the PMMA/PZT coating. Additionally, periodically-excited specimens generated electrical potential. The highest value of the harvested electrical energy was registered in the PS/PZT microresonator (up to 3 mV). The lowest result was 2.1 mV, registered for PVB/PZT, and PMMA/PZT generated up to a 2.5-mV voltage.

Investigation of the mechanical properties showed that there was a very slight difference in Martins and Vickers microhardness between the different coatings, i.e., HM differed by about 17 N/mm^2^ and HV only by ~3. However, the microresonators’ film with PMMA/PZT showed more elastic deformation than the other materials. The scratch test showed that the PMMA/PZT coating was almost twice as hard compared to the other two, i.e., the average critical load was 3.988 N, meaning a very resistant coating to the scratch damage. The PVB/PZT coating was rather soft and not that resistant with a critical load of 1.865 N. The PS/PZT average critical load was 2.556 N. Moreover, the vibrational amplitude and Young’s modulus were investigated for all three binders. The registered results were 6.3 GPa and 164 nm, 5.3 GPa and 366 nm, and 3.9 GPa and 100 nm for the PMMA, PS, and PVB binders, respectively.

Adding the PMMA/PZT layer with the formed periodical microstructure to the proposed microresonator increased its accuracy due to the possibility to use both electrical and optical measurement techniques. The added nanocomposite allowed controlling not only the mechanical properties of the microresonator, but the optical parameters, as well. The change of the period by 10 nm followed a 0.1% diffraction efficiency change and a 0.15° shift of the diffraction angle.

Finally, it may be stated that PMMA and PS binding materials should be selected in order to achieve the highest quality factors in the design of microresonators. Using the designed system in microfluidics it might unlimit the manipulation of small liquid plugs in microchannels, to increase flow of high viscosity fluids, and when excited, to reduce air bubbles between two liquid plugs.

## Figures and Tables

**Table 1:** Chemical characterization of the PVB/PZT sample.

Element	Series	The Unnormalized Concentration in Weight Percent of the Element (wt.%)	The Normalized Concentration in Weight Percent of the Element (norm. wt.%)	The Atomic Weight Percent (norm. at.%)	An Error in the Weight Percent Concentration (%)	A Map of the Distribution and Relative Proportion (Intensity)
Carbon	K-series	45.31	42.24	61.36	5.51	Figure 7a
Oxygen	K-series	34.11	31.81	34.68	33.64	Figure 7b
Titanium	K-series	3.59	3.35	1.22	0.13	Figure 7c
Zirconium	L-series	8.38	7.82	1.49	0.36	Figure 7d
Lead	L-series	15.86	14.79	1.24	0.57	Figure 7e
	Sum:	107.25	100	100		

**Table 2:** Chemical characterization of the PMMA/PZT sample.

Element	Series	The Unnormalized Concentration in Weight Percent of the Element (wt.%)	The Normalized Concentration in Weight Percent of the Element (norm. wt.%)	The Atomic Weight Percent (norm. at.%)	An Error in the Weight Percent Concentration (%)	A Map the Distribution and Relative Proportion (Intensity)
Carbon	K-series	41.30	37.38	55.88	5.07	Figure 8a
Oxygen	K-series	39.29	35.56	39.90	34.41	Figure 8b
Aluminum	K-series	0.15	0.14	0.09	0.04	Figure 8c
Titanium	K-series	3.50	3.17	1.19	0.13	Figure 8d
Zirconium	L-series	8.89	8.05	1.58	0.38	Figure 8e
Lead	L-series	17.36	15.71	1.36	0.62	Figure 8f
	Sum:	110.49	100	100		

**Table 3:** Chemical characterization of the PS/PZT sample.

Element	Series	The Unnormalized Concentration in Weight Percent of the Element (wt.%)	The Normalized Concentration in Weight Percent of the Element (norm. wt.%)	The Atomic Weight Percent (norm. at.%)	An Error in the Weight Percent Concentration (%)	A Map the Distribution and Relative Proportion (Intensity)
Carbon	K-series	77.20	69.40	87.27	8.91	Figure 9a
Oxygen	K-series	11.85	10.65	10.05	19.44	Figure 9b
Titanium	K-series	2.95	2.65	0.84	0.12	Figure 9c
Zirconium	L-series	6.91	6.21	1.03	0.31	Figure 9d
Lead	L-series	12.32	11.08	0.81	0.46	Figure 9e
	Sum:	111.23	100	100		

**Table 4:** Gauge factor (GF).

Value	PVB/PZT Coating	PMMA/PZT Coating	PS/PZT Coating
GF	−139.5	1151.0	−3458.8

**Table 5:** Capacitance values of microresonators.

Coating	Capacitance C, pF
PVB/PZT	0.13
PMMA/PZT	0.2
PS/PZT	0.1

**Table 6:** Main properties of the designed microresonators.

Coating	Binding Material	Density, ρ, kg/m^3^	Damping Ratio, ζ	Frequency, f, Hz	Natural Frequency, ω_n_, rad/s	Young Modulus, E, GPa
PVB/PZT	PVB	6298	0.0052 ± 0.0003	26.5 ± 0.4	166 ± 2.8	3.9 ± 0.3
PMMA/PZT	PMMA	6316	0.0077 ± 0.0006	29.4 ± 0.9	185 ± 5.8	6.3 ± 0.8
PS/PZT	PS	6288	0.0080 ± 0.0004	28.3 ± 0.6	178 ± 4.0	5.3 ± 0.4

**Table 7:** Sheet resistivity values.

Coating	Sheet Resistivity, (Ω/sq)
PVB/PZT	>20,000
PMMA/PZT	>20,000
PS/PZT	>20,000

**Table 8:** Microhardness measurement results.

	PVB/PZT			PMMA/PZT			PS/PZT		
	HM	HV	h_max_	HM	HV	h_max_	HM	HV	h_max_
*n* = 10	N/mm^2^		μm	N/mm^2^		μm	N/mm^2^		μm
**X.**	320.81	33.87	4.32	337.40	35.77	4.34	330.22	32.54	4.43
**q**	108.81	12.11	0.18	84.87	9.58	0.13	81.26	7.81	0.10
**s**	152.07	16.93	0.25	118.61	13.40	0.18	113.56	10.91	0.14
**V/%**	47.40	49.98	5.76	35.15	37.45	4.16	34.39	33.54	3.20
**Min.**	108.60	11.90	4.10	133.80	14.90	4.00	132.50	12.00	4.27
**Max.**	515.00	55.30	4.95	567.40	61.30	4.61	514.30	47.90	4.67
**R**	406.38	43.90	0.85	433.59	46.33	0.61	381.74	35.87	0.40
**R/%**	126.67	128.12	19.73	128.51	129.51	14.04	115.60	110.24	9.08

Here, HM, microhardness in the Martins scale; HV, microhardness in the Vickers scale; *n*, number of single readings per block; X., means value of the individual mean values, the so-called arithmetic mean value; q, a random measurement error (within a certain probability (confidence level), the likely ”true” value m of the measured quantity is in an interval (two-sided confidence interval) around the measured mean value X of a measurement series, and the borders of this interval have the distance q from the “true” value); s, the standard deviation, a measure for the deviation of the spread of the single readings of a measurement series around their common mean value X; V/%, the coefficient of variation describing the spread of a measurement series in percent; Min., the lowest reading of the block; Max., the highest reading of the block; R, the range, the difference between the highest and the lowest reading of the block; R/%, the range in percent.

**Table 9:** Critical load values of microresonators.

Coating	Critical Load 1, N	Critical Load 2, N	Critical Load 3, N	Critical Load 4, N	Average Critical load, N
PVB/PZT	1.88	1.89	1.76	1.93	1.865
PMMA/PZT	4.04	3.91	3.97	4.03	3.988
PS/PZT	2.48	2.67	2.55	2.53	2.556
